# Low-Cost Gas Sensors Produced by the Graphite Line-Patterning Technique Applied to Monitoring Banana Ripeness

**DOI:** 10.3390/s110606425

**Published:** 2011-06-17

**Authors:** Alexandra Manzoli, Clarice Steffens, Rafaella T. Paschoalin, Alessandra A. Correa, William F. Alves, Fábio L. Leite, Paulo S. P. Herrmann

**Affiliations:** 1 National Nanotechnology Laboratory for Agribusiness (LNNA), Embrapa Instrumentation, P.O. Box 741, 13560-970, São Carlos, SP, Brazil; E-Mails: alexandra@cnpdia.embrapa.br (A.M.); clarice@cnpdia.embrapa.br (C.S.); rafa_rrtp@hotmail.com (R.T.P.); wfa23@yahoo.com.br (W.F.A.); 2 Center for Engineering, Modeling and Applied Social Sciences (CECS), Federal University of ABC (UFABC), 09210-170, Santo André, SP, Brazil; E-Mail: alealvescorrea@gmail.com; 3 Center Multidisciplinary, Federal University of Acre (UFAC), 69915-900 Cruzeiro do Sul, AC, Brazil; 4 Department of Physics Mathematics and Chemistry (DFMQ), Federal University of São Carlos (UFScar), 18052-780, Sorocaba, SP, Brazil; E-Mail: fabioleite@ufscar.br

**Keywords:** electronic nose, conducting polymer, low-cost gas sensors

## Abstract

A low-cost sensor array system for banana ripeness monitoring is presented. The sensors are constructed by employing a graphite line-patterning technique (LPT) to print interdigitated graphite electrodes on tracing paper and then coating the printed area with a thin film of polyaniline (PANI) by *in-situ* polymerization as the gas-sensitive layer. The PANI layers were used for the detection of volatile organic compounds (VOCs), including ethylene, emitted during ripening. The influence of the various acid dopants, hydrochloric acid (HCl), methanesulfonic acid (MSA), *p*-toluenesulfonic acid (TSA) and camphorsulfonic acid (CSA), on the electrical properties of the thin film of PANI adsorbed on the electrodes was also studied. The extent of doping of the films was investigated by UV-Vis absorption spectroscopy and tests showed that the type of dopant plays an important role in the performance of these low-cost sensors. The array of three sensors, without the PANI-HCl sensor, was able to produce a distinct pattern of signals, taken as a signature (fingerprint) that can be used to characterize bananas ripeness.

## 1. Introduction

Estimation of the degree of maturity of fruit at harvest is of great importance because physiologically immature fruits have not completed their growth, while fruit harvested at a far more advanced stage toward or after maturity are more susceptible to disease and deteriorate rapidly [1]. Assessment and monitoring of food quality, during both production and storage is of prime importance to enhance its quality at the point of sale. With the aim of minimizing production losses and also preserving the unique features of each fruit, a strategy involving electronic artificial nose systems that use sensors to monitor volatile organic compounds (VOCs) emitted during fruit ripening has been reported [2]. These sensors are non-specific, since their response is an overall profile of the response to the various chemical species present in the VOCs, including ethylene. This behavior is known as global selectivity [2].

An electronic nose consists of a mechanism for chemical detection, such as an array of electronic sensors, and a mechanism for pattern recognition. Artificial electronic nose systems can recognize specific chemical compounds, even in a complex mixture of vapors, by a rapid test [3]. However, many of these sensors are produced by sophisticated and expensive technology such as lithography [4,5].

The use of sensors based on conductive polymers in electronic noses is very promising. Polyaniline (PANI) is highly stable under ambient conditions and also has a wide range of properties, such as high electrical conductivity, redox reversibility and ease of synthesis and doping in aqueous solution [6–8]. Interest has focused upon the discovery that acids with certain functional groups may be used to render the emeraldine salt (ES) form of the polymer processable from solution [9]. One of the main developments is the combination of PANI with some dopants. It has been shown that particular dopants can affect strongly the morphology and electrical properties of thin films used in sensors [10–15].

As far as we know, there are only a few studies in the literature in which sensors were used in an electronic nose to analyze the state of banana ripeness [2,16]. Llobet *et al.* used commercially available metal oxide sensors [2]. In comparison with these metal oxide sensors, conducting polymer sensors have many advantages, such as high sensitivity and short response time. The polyaniline molecular chain structure can be modified by doping, making it soluble in certain solvents and increasing their sensitivity [17]. Steffens *et al.* developed a gas sensor based on interdigitated line patterns of graphite on tracing paper [16]. Sensors were coated with a thin film of PANI by *in-situ* polymerization, rapid expansion of polyaniline in pressurized fluid and precipitation of polyaniline with pressurized fluid.

In the present study, low-cost gas sensors were constructed by a line-patterning technique (LPT) [18], used to deposit interdigitated graphite electrodes [19] on tracing paper, which was then coated with a thin film of PANI formed by *in-situ* polymerization [20]. The influence of several acid dopants (HCl, MSA, TSA and CSA) on the performance of these sensors was investigated. The sensors (“electronic noses”) doped with these acids were used to detect the gases emitted during banana ripening. The degree of doping in the PANI films was investigated by UV-Vis spectroscopy and sensor performance was studied by measuring their electrical properties.

## 2. Experimental Section

Water was purified in a Milli-Q system (Millipore Co., MA, USA) to a nominal resistivity of 18.2 MΩ cm^−1^. High-purity N_2_ was purchased from White Martins Special Gases. Aniline (Aldrich, St. Louis, USA) was distilled before use. Hydrochloric (HCl), methanesulfonic (MSA), toluenesulfonic (TSA) and camphorsulfonic (CSA) acids, ammonium hydroxide and ammonium persulfate (Merck, Darmstadt, Germany) were of analytical grade. A commercial aqueous graphite paste, Aquadag E (Acheson Co., ON, Canada), was utilized. Tracing paper (weight 63 g/m^2^) was purchased from local stationers. Three sets of unripe bananas (*Musa cavendish*) were purchased from a local supermarket.

**Figure 1.**
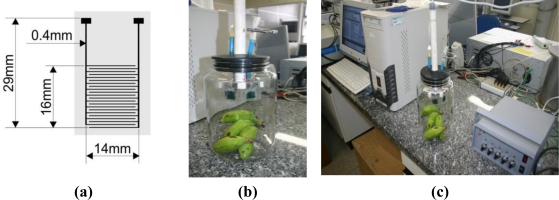
**(a)** Interdigitated electrodes in 11 pairs (the drawing is unscaled), **(b)** chamber with the bananas and **(c)** whole system (computer, chamber and electronic circuit).

Thin films of PANI-HCl were deposited, by *in-situ* polymerization [20], on interdigitated graphite electrodes printed over a rectangular area of 16 mm by 14 mm. These consisted of interdigited electrodes in 11 parallel pairs, 0.25 mm wide, separated by 0.5 mm gaps [see Figure 1(a)], prepared on tracing paper by LPT, as described previously [18]. These sensors were dedoped by immersion in 0.1 M NH_4_OH solution for 30 s (to form PANI-EB) and then redoped for 90 s with a 0.1 M solution of the desired acid dopant. These conditions were chosen after optimization. The degree of doping of the PANI was investigated with a Shimadzu UV-1601 PC UV-VIS spectrometer. The electrical resistance in air of the uncoated interdigitated electrodes of graphite and of PANI sensors doped with various acids was measured with a commercial multimeter (Fluke).

Each of the four sensors in the array was doped with one of the following acids: HCl, MSA, TSA, CSA, The electrical connections of the sensor array consisted of electromechanical contacts with two metal plates made of aluminum. This array was tested for the detection of the volatile organic compounds (VOCs) emitted during whole banana ripening. Unripe bunches of bananas (*Musa cavendish*) were initially used for collecting the experimental data because all bananas of a given bunch ripen simultaneously during measurements. The criterion of ripeness was the color of the banana peel. The array of sensors was kept at ambient conditions for 30 min before beginning measurement. Afterwards, the electronic nose composed of the array of four sensors was introduced into the 4.5 L chamber containing the green bananas and the data was acquired each 5 min for 84 h. Figure 1(b) shows the sensor array inside the chamber with the bananas and Figure 1(c) illustrates the whole system (computer, chamber and electronic circuit). This experiment was performed in triplicate, using a fresh array of sensors and bunch of bananas for each repetition. The glass chamber has a hole of 0.3 cm radius, which allows gas exchange with surrounding air. The temperature and humidity inside the chamber were measured every five minutes by a thermohygrometer (MINIPA MT-241).

**Figure 2.**
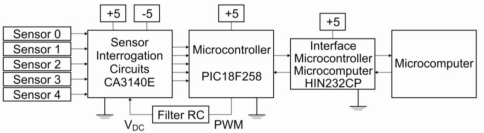
Block diagram of the electronic system of the electronic nose.

The electronic system consisted of five independent two-terminal voltmeters working in parallel, with the gain set manually to allow optimization of the analog-digital conversion range. A pulsed constant current was supplied to each sensor through a pulse-width modulated current source (operational amplifier feedback network) of frequency 1.0 KHz, adjusted to set the baseline of each sensor (in lab air) to the same value of 400 mV. The electronic system was developed with microcontroller 16F88. This microcontroller has a pulse width modulator (PWM) to generate an mV mode. The frequency range of the PWM is 1.22 kHz to 203.8 kHz (Figure 2). The whole system—electronic circuit, low-cost gas sensors, 4.5 L chamber and computer software—is described as a low-cost electronic nose. Since the current through each sensor is kept constant throughout the experiment (see Results for current values), the voltage across the sensor is proportional to its resistance by Ohm’s law. Multivariate analysis was carried out by Principal Component Analysis (PCA), a statistical method to reduce dimensionality in a way that retains as much as possible of the data set variation. The first principal component, PC 1, has the largest possible variance, PC 2 the second largest and so on [21].

To test the performance (sensitivity and reversibility η (%)) of the gas sensors doped with the acids at humidity, the baselines were adjusted to the same voltage of 400 mV in air. These measurements were made with the purpose of charactering the sensors in relation to humidity, since the flow of nitrogen removes humidity from the chamber. The sensors were then exposed to laboratory air (60% relative humidity, 25 °C) for 10 min, then for 10 min to a flow of dry N_2_ at 1,000 mL min^−1^ inside the 4.5 L chamber, after which this cycle was repeated several times. Data acquisition was taken every 1 min for 100 min. The sensitivity [S (%)] and reversibility [η (%)] of the sensors were calculated from Equations (1) and (2) [12]:

(1) $$S_{\%}=\frac{\left(V-V_{o}\right)}{V_{o}}*100$$

(2) $$\eta_{\%}=\frac{\left(V-V_{f}\right)}{\left(V-V_{o}\right)}*100$$

where V_0_ is the initial voltage of the sensor (in air), V the voltage after exposure to a flow of dry N_2_ and V_f_ the minimum voltage reached in the presence of laboratory air after the exposure to dry N_2_.

## 3. Results and Discussion

**Figure 3.**
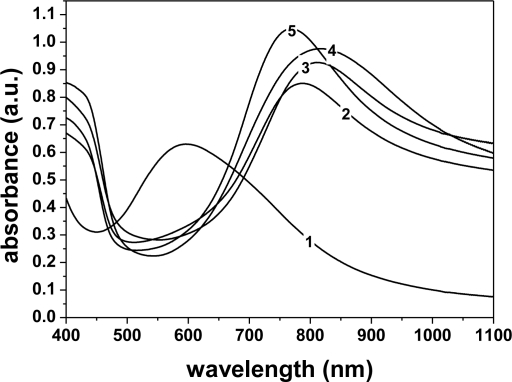
UV/Vis absorption spectra of thin films of PANI (1) dedoped in NH_4_OH solution (EB) and of thin film of polyaniline emeraldine salt doped with various acids: (2) CSA, (3) TSA, (4) HCl and (5) MSA, on PET.

Figure 3 shows the visible absorption spectra for PANI doped with the various dopant acids, with the PANI-emeraldine base (EB) spectrum as a control. In the EB spectrum (curve 1), one band was observed at 600 nm (n-π* transition). The doping of PANI-EB with various acids suppressed this band, since this absorption is related to transitions in the quinoid rings, which are no longer present in the ES (salt) form, as they are converted into poly(semiquinone) cation radicals [22]. In the doped PANI films, two bands were seen, which are related to polaron absorption around 420 nm and 800 nm [815 nm (HCl), 810 nm (TSA), 787 nm (CSA), 770 nm (MSA)], indicating that the samples are in the conducting state [23]. The small differences in the doping level (peak shifts) may be attributed to the higher or lower degree of protonation of the polymers and also to some variation in the molecular conformation [10]. By comparing the five spectra in Figure 3, it is seen that the PANI doped with HCl (curve 4) and TSA (curve 3) showed a higher degree of doping than PANI doped with CSA (curve 2) and MSA (curve 5). It is important to investigate the microstructural alteration of the films by different dopant anions, since these have a strong influence on the electroactivity and conductivity of the gas sensor coated with PANI film.

In light of these results, arrays of the four doped polymer sensors were employed in the design of an electronic nose, which was used to monitor the ripeness of bananas. In these experiments, the temperature was 25–27 °C, the relative humidity 43–81%, and the fruit samples weighed 500 grams.

**Figure 4.**
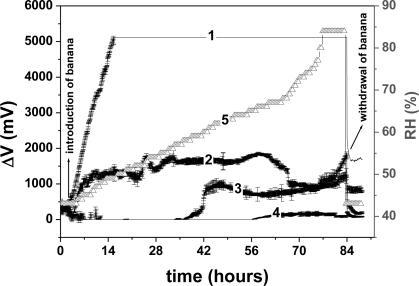
Average response of three arrays of sensors to the volatiles emitted during banana ripening. Each of the four sensors in the array was doped with one of the following acids: (1) HCl, (2) CSA, (3) TSA, (4) MSA and (5) relative humidity.

Figure 4 presents the average responses of the three arrays of sensors to the volatile organic compounds (VOC) emitted during banana ripening. The data were acquired as voltage variation (ΔV) *versus* time. In this figure, the voltage values begin at about 0 V because the baseline was subtracted.

As the electrical resistance of the uncoated interdigitated electrodes was greater than 40 MΩ, the current through the tracing paper may be considered negligible. The resistances of the PANI sensors with the four different dopants were: HCl 5.0 ± 0.3; TSA 12.3 ± 0.4; CSA 20.2 ± 0.5; MSA 22.7 ± 0.1 kΩ. These measurements were carried out in the lab air, before placing the sensors in the chamber of the electronic nose. In the chamber, the baseline of each sensor was adjusted to the same value of 400 mV. Thus, the current values applied to each sensor were: HCl 80.0 μA, TSA 32.0 μA, CSA 19.8 μA and MSA 17.6 μA. The power dissipated in the array of sensors varied from 6.8–32 × 10^−6^ W, insufficient to cause any Joule heating in the sensitive layers.

In Figure 4, it can be seen that the four sensors of the array differed widely in their levels of sensitivity to the VOCs emitted during banana ripening. Thus, their response must be associated with the type of dopant used, and each dopant may provide a type of selectivity to the sensor, known as molecular recognition [13]. Films of PANI doped with different acids behaved differently in the presence of the same substance, with distinct outputs for each film used, as reported by MacDiarmid [24]. Note that the three arrays of sensor presented similar data.

The volatiles emitted in banana ripening are usually complex mixtures. Mayr *et al.* [25] conducted an extensive study employing proton reaction mass spectrometry, and identified quite a large number of volatile fractions (∼250 compounds) at various stages of banana ripeness. This complexity makes it almost impossible to find sensors corresponding to every individual component of the gas mixture.

**Figure 5.**
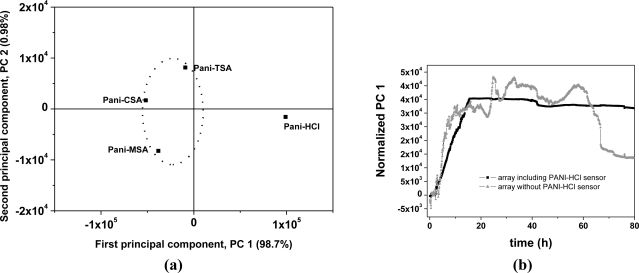
**(a)** PCA chart of first two principal components and **(b)** normalized PC1 against the time.

By employing PCA [Figure 5(a)], it was possible to differentiate and to group the dopants used in the sensors with respect to the levels of sensitivity to the VOCs emitted during ripening. An interesting observation was that the sensor doped with HCl showed characteristics that separated it from the other sensors on the PCA chart. Figure 5(b) presents the first principal component (PC 1), with and without the PANI-HCl sensor in the analysis, plotted against time. It can be seen in Figure 5(b) that the sensor array without the PANI-HCl sensor was able to produce a distinct temporal pattern, taken as a signature (fingerprint) that could be used to monitor banana ripening. This profile indicates the initial maturation stage, characterized by a low production of carbon dioxide or oxygen absorption, the plateau showing ethylene production up to the 58 h and, after this, the final stage of declining signal. These features are characteristic of the standard banana climacteric phase of accelerated respiration [26]. Thus, this signature pattern correlates the sensor data with the state of ripeness of the banana. The oscillations observed in the PC 1 curve produced without the PANI-HCl sensor might be due to variations in compounds and their concentrations during the banana ripening. The array of sensors with PANI-HCl showed a sharp initial response in PC 1 with saturation after 14 h, when the electronic circuit reaches its upper limit. The electronic circuit used in this nose has a maximum dc output voltage of 5 V and above this limit it is incapable of detecting other volatiles emitted during the remaining maturation period (see curve 1, Figure 4) [16]. Saturation does not happen with the other sensors, doped with CSA, TSA and MSA, as can be seen in Figure 4.

To understand the distinct behavior of the sensor of PANI doped with HCl, it is interesting to compare the resistances in air of the PANI films doped with the four different acids, reported earlier. These values increased with the type of acid in the following order: HCl < TSA < CSA < MSA. The highest level of doping, observed in PANI-HCl, associated with the lowest resistance (less than half that of TSA), explains why this sensor gave a steep initial response to the volatiles emitted at the start of banana ripening (see Figure 4). For the development of these sensors, further research will be required to identify and measure the many interfering gases emitted during the ripening process.

**Figure 6.**
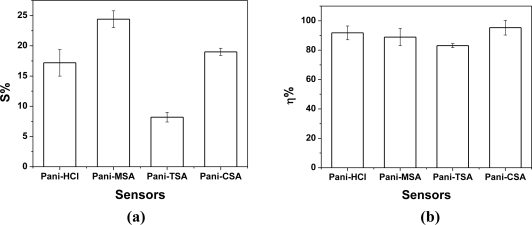
**(a)** Sensitivity (%) and **(b)** reversibility (%) of sensors based on thin film PANI-ES prepared by *in-situ* polymerization and doped by anion replacement with various acids, on exposure to dry nitrogen flow; (I) standard deviation of three experiments.

The sensitivity [Figure 6(a)] and reversibility [Figure 6(b)] of the four sensors in the array were measured in triplicate, at 25 °C, by exposure to dry nitrogen flowing through the chamber, alternating with air at 60% relative humidity. These results were important to evaluate the speed of electrical response of the device and the interaction of the sensor with humidity and also to characterize the sensors with respect to reversibility. The sensitivity was different for each of the sensors, varying from 8.2% to 24.4% [Figure 6(a)], as discussed above. In Figure 6(b), all the sensors tested were observed to have excellent reversibility. These results indicate that thin film PANI-ES prepared by *in-situ* polymerization is a promising material for technological applications such as the development of gas sensors.

## 4. Conclusions

Graphite patterns were successfully produced on a tracing paper substrate by LPT, forming interdigitated electrodes. The graphite lines were adequately coated with PANI thin film by *in-situ* polymerization to produce low-cost gas sensors.

These sensors were successfully dedoped and redoped with various acid dopants. The sensors then exhibited changes in electrical resistance responses upon exposure to dry nitrogen and to VOCs from ripening bananas, which depended on the dopant used. These results open up the new possibility of arrays of these sensors being used as electronic noses. The responses of sensors doped with CSA, TSA and MSA in the monitoring of banana ripening indicate their potential for application as an electronic nose to practical technological problems. The development of this low-cost apparatus will provide a more efficient monitoring of the maturation of fruits, bringing consumers better quality products.
